# Changes in the Calcium-Parathyroid Hormone-Vitamin D Axis and Prognosis for Critically Ill Patients: A Prospective Observational Study

**DOI:** 10.1371/journal.pone.0075441

**Published:** 2013-09-20

**Authors:** Jieyu Hu, Zuojie Luo, Xiaoqin Zhao, Qiang Chen, Zhaoyan Chen, Hua Qin, Yingfen Qin, Xinghuan Liang, Yingjun Suo

**Affiliations:** 1 Intensive Care Unit, First Affiliated Hospital of Guangxi Medical University, Nanning, China; 2 Department of Endocrinology, First Affiliated Hospital of Guangxi Medical University, Nanning, China; University of Tennessee, United States of America

## Abstract

**Objective:**

Vitamin D deficiency is prevalent in critically ill patients and may contribute to suboptimal clinical outcomes, but little is known about alterations of the calcium-parathyroid hormone (PTH)-vitamin D axis and prognosis in these individuals.

**Methods:**

A prospective observational study was conducted on 216 patients admitted to a university-affiliated, tertiary-care medical intensive care unit(MICU) between June 2011 and December 2012. Serum levels of 25-hydroxyvitamin D, ionised calcium and intact PTH were determined within 24 h of MICU admission. The primary end point was all-cause hospital mortality within 90-days of admission.

**Results:**

95 patients (44%) showed 25-hydroxyvitamin D deficiency. Patients deficient in vitamin D showed significantly higher Acute Physiology and Chronic Health Evaluation II (APACHE II) score, rate of positive blood culture, incidence of multiple organ dysfunction syndrome, and 90-day mortality rate than did patients with vitamin D insufficiency or sufficiency (*P*<0.05), as well as lower levels of serum IgG. 25-Hydroxyvitamin D deficiency was identified as an independent risk factor for mortality (OR = 3.018, 95%CI 1.329–6.854, *P* = 0.008). Hypovitaminosis D in PTH-responders was associated with higher mortality than was the same condition in non-responders (*P*<0.05).

**Conclusions:**

These results suggest that vitamin D deficiency is prevalent among MICU patients, suggesting a significant derangement of the calcium-PTH-vitamin D axis in critically ill patients. Vitamin D deficiency is an independent risk factor for 90-day mortality, and hypovitaminosis D in PTH-responders is associated with higher mortality than is the same condition in non-responders.

## Introduction

Vitamin D is now recognised as playing an important role not only in the regulation of calcium metabolism and bone health [1] but also in numerous additional processes in the immune system, in endothelial and mucosal tissues, and in glucose metabolism [2], [3]. In fact, vitamin D receptors are expressed in nearly all tissues and cells of the human body. Vitamin D deficiency, the most frequent medical condition in the world [1], has been associated with myocardial infarction, diabetes, autoimmune disease, chronic obstructive pulmonary disease, tuberculosis, and excess mortality in the general population [4]. A meta-analysis of 32 randomized controlled trials involving 74,789 participants, primarily elderly women, found that vitamin D_3_ supplementation decreased all-cause mortality by 6% [5].

Critically ill patients are at high risk of progressive vitamin D deficiency because they are less likely than other populations to receive adequate exposure to sunlight and adequate amounts of vitamin D in their diet [4]. Incidence of vitamin D deficiency among critically ill patients has been estimated at 23–82% [6], [7], [8], . This deficiency in critically ill patients is associated with several morbidities, including severe hypocalcaemia, hyperglycaemia, organ dysfunction, and increased susceptibility to nosocomial infection. Important gaps remain in our understanding of the effects of vitamin D deficiency on critically ill patients and their prognosis. For example, several studies have associated vitamin D deficiency with increased mortality among intensive care unit (ICU) patients [8], [10], [11], but other studies have found no such association [9], [12]. In addition, although calcium, parathyroid hormone (PTH) and vitamin D are known to interact, little is known about how this axis is altered in critical illness. Given the prevalence of vitamin D deficiency in this vulnerable group of patients, understanding perturbations in this axis is important for optimising treatments.

The present study sought to determine the prevalence of vitamin D deficiency among critically ill adults, characterise changes in the calcium-PTH-vitamin D axis and assess the effects of these changes on disease severity and prognosis. Serum 25-hydroxyvitamin D [25(OH)D] level served as the indicator of vitamin D status.

## Materials and Methods

### Study Design and Population

We performed a prospective observational study of 216 patients admitted to the medical intensive care unit (MICU) of the First Affiliated Hospital of Guangxi Medical University in Nanning, China (northern latitude 22°49′) between June 2011 and December 2012. All patients 18 years or older who stayed more than 48 h in the MICU were included, as long as they did not satisfy any of the following exclusion criteria: age below 18 years, ongoing pregnancy, current breastfeeding, chronic kidney disease, parathyroid disease, fluid resuscitation, acute haemodilution, vitamin D supplementation prior to admission, or treatment with medications known to affect vitamin D or calcium metabolism.

The study was approved by the Research Review Board of the First Affiliated Hospital, according to the Declaration of Helsinki. Informed consent was signed from the patient’s legal guardians at the time of MICU admission.

### Data Collection and Definitions

The primary end point was hospital mortality within 90 days of ICU admission. Data were collected at baseline on demographics, diagnosis, score on the Acute Physiology and Chronic Health Evaluation II (APACHE II), and basic haematological and biochemical parameters. The APACHE II score for each patient was taken to be the worst score obtained within 24 h of MICU admission. Blood was drawn within 24 h of MICU admission, and levels of serum 25(OH)D, ionised calcium (iCa) and intact parathyroid hormone (iPTH) were determined. Levels of 25(OH)D were measured by high-performance liquid chromatography (HP1100, Agilent, USA; interassay CV 4.1–5.3%) and iPTH by radioimmunoassay (DiaSorin, Italy; interassay CV 7–9%), ionised calcium was determined with the GEM Premier 3000 critical care analyzer (interassay CV 0.9–1.8%).

Vitamin D status was determined based on the 25(OH)D level measured within 24 h of MICU admission. A level of 0–19.9 ng/ml (0–49.9 nmol/L) was defined as deficiency, 20–29.9 ng/ml as insufficiency, and ≥30 ng/ml as sufficiency [13]. Hypovitaminosis D was defined as a 25(OH)D level <30 ng/ml. Secondary hyperparathyroidism was defined as a serum iPTH level >75 pg/ml, corresponding to the upper limit of the laboratory reference range [14]. Hypocalcaemia was defined as an iCa level <1.15 mmol/L.

To evaluate changes in the calcium-PTH-vitamin D axis, patients were classified as PTH-responders or non-responders. PTH-responders were defined as patients who presented elevated iPTH (>75 pg/ml) together with hypovitaminosis D (<30 ng/ml) and/or ionised hypocalcaemia (iCa <1.15 mmol/L) based on measurements made within 24 h of MICU admission. Patients presenting with both impaired PTH response (≤75 pg/ml) and hypovitaminosis D were categorised as PTH non-responders.

### Statistical Analysis

Data were analysed using SPSS17 (IBM, Chicago, IL, USA). Continuous variables with a normal distribution were expressed as means ± SD; otherwise, they were expressed as medians with the first and third interquartile ranges (IQR1, IQR3). Categorical variables were reported as percentages. Differences between groups were assessed using the chi-squared test for categorical variables and Student’s t-test or non-parametric test for continuous variables. Coefficients of partial correlation between variables were calculated using Pearson’s or Spearman’s analysis, depending on whether the data for the variables were normally distributed. Multivariate logistic regression was used to identify independent predictors of 90-day mortality. Two-sided *p*-values <0.05 were considered statistically significant.

## Results

### Patient Characteristics

A total of 216 adult patients admitted to the MICU met our inclusion criteria during the study period (Table S1). The most frequent reasons for exclusion were an MICU stay <48 h, chronic renal failure, and fluid resuscitation. All 216 patients were xanthoderms; 120 (55.6%) were men, and 96 (44.4%) were women. Median age was 64 years (IQR1 50, IQR3 75) with a median APACHE II score of 21 (18, 26). The most frequent primary admission diagnoses were severe pneumonia with respiratory failure (47.2%), acute exacerbations of chronic obstructive pulmonary disease (19%) and intra-abdominal infection (15.3%). Most patients (185, 85.6%) required mechanical ventilation, and all-cause 90-day hospital mortality was 28.7% for the entire study population.

### Vitamin D Status at MICU Admission

Of the 216 patients studied, 95 (44%) were characterised as deficient in 25(OH)D, 58 (26.8%) as insufficient, and 63 (29.2%) as sufficient. The lowest and highest observed levels of serum 25(OH)D were 11.2 and 55.7 ng/ml. No seasonal variability was observed in 25(OH)D levels. Characteristics of patients stratified by vitamin D status at admission are shown in Table S2.

Patients deficient in 25(OH)D had a higher APACHE II score (*P*<0.001), iPTH level (*P*<0.001), positive blood culture rate (*P* = 0.015), incidence of multiple organ dysfunction syndrome (MODS) (*P* = 0.013) and 90-day mortality (*P* = 0.003) than did patients insufficient or sufficient in 25(OH)D. Patients deficient in 25(OH)D also had lower levels of Ig G (*P*<0.001) and iCa (*P*<0.001) than did the other two groups.

The three groups did not significantly differ in age, sex, length of ICU stay, number of days on ventilation, or levels of total calcium, serum phosphate or albumin. Patients deficient/insufficient in vitamin D showed a trend towards higher CRP levels than those sufficient in vitamin D, but this difference was not statistically significant.

### Calcium-PTH-vitamin D Axis

Most patients (89.4%) presented at MICU admission with ionised hypocalcaemia, defined as iCa <1.15 mmol/L. Secondary hyperparathyroidism, defined as iPTH >75 pg/ml, was present in 56.6% of hypocalcaemic patients and 59.5% of hypovitaminosis D patients. PTH-responders had higher iCa levels than did non-responders (*P*<0.001), but the two groups did not differ in levels of total calcium or 25(OH)D (*P*>0.05; Table S3).

### Relationships among Components of the Vitamin D Axis

Levels of 25(OH)D at MICU admission negatively correlated with iPTH levels (*r* = −0.232, *P* = 0.001), but they did not correlate with levels of iCa (*r* = 0.12, *P* = 0.152) or total calcium (*r* = 0.143, *P* = 0.108). Levels of iPTH at admission strongly correlated with levels of iCa (*r* = 0.682, *P* = 0.0001) and total calcium (*r* = 0.468, *P* = 0.0001).

### Associations between Vitamin D Status and Illness Severity and Outcomes

Levels of 25(OH)D at admission negatively correlated with APACHE II score (*r* = −0.325, *P*<0.001) and hospital mortality (*r* = −0.276, *P*<0.001). Conversely, 25(OH)D level correlated positively with Ig G level (*r* = 0.31, *P* = 0.002). In contrast, levels of iPTH, iCa and total calcium did not show any relationship with APACHE II score or hospital mortality (*P*>0.05).

Multiple logistic regression in which vitamin D status was treated as a categorical variable identified APACHE II score, 25(OH)D deficiency, plasma lactate and age as independent predictors of 90-day mortality. Neither iPTH nor iCa was associated with mortality (Table S4). In addition, hypovitaminosis D in PTH-responders was associated with higher APACHE II score and mortality than was the same condition in non-responders (*P*<0.05, Table S3).

## Discussion

Our results demonstrate a high prevalence of hypovitaminosis D in the critically ill patient population, with vitamin D deficiency observed in 44% of the patients in this study. Vitamin D deficiency was associated with higher disease severity and hospital mortality. It was also associated with higher incidence of positive blood culture and of MODS, as well as lower Ig G levels, all of which may worsen the prognosis of ICU patients.

Although our patients deficient in vitamin D fared worse than did our patients with insufficient or sufficient vitamin D for nearly all outcomes examined, the deficient group nevertheless did have a shorter ICU stay and fewer days on the ventilator than did the sufficient group. This is in contrast to recent work by Matthews [10] showing that the deficient group stayed longer in the ICU. This discrepancy may be due to the different type of study population: our study examined all-cause MICU patients, whereas Matthews et al. examined specifically surgical ICU patients. The discrepancy may also be due to high mortality among our patients deficient in vitamin D soon after MICU admission, resulting in overall shorter ICU stay and fewer days on the ventilator.

To our knowledge, ours is the first study to report a positive correlation between serum 25(OH)D concentration and Ig G levels in an ICU population. This relationship contrasts with the inverse relationship observed in chronic diseases that often involve vitamin D deficiency, such as systemic lupus erythematosus (SLE) [15] and cystic fibrosis [16]. In fact, cellular studies have shown that 1,25(OH)_2_D, the active form of vitamin D, decreases B cell proliferation, plasma cell differentiation and Ig G secretion [17]. Our observational study does not allow us to discern whether serum 25(OH)D levels are causally linked to Ig G levels. Further studies are needed to examine the correlation between these two factors in both critical illness and chronic disease.

Although serum levels of 25(OH)D are widely used as an indicator of vitamin D status, consensus is lacking about the cut-off values for defining deficiency and sufficiency. Serum 25(OH)D levels are inversely associated with PTH levels until the former reach 30–40 ng/ml, at which point PTH levels begin to level off [18], [19] and intestinal calcium absorption is maximal [20]. Therefore most experts suggest defining vitamin D sufficiency as serum 25(OH)D ≥30 ng/ml and deficiency as <20 ng/ml [1]. Our study adopted these thresholds to define vitamin D status in critically ill patients.

Hypovitaminosis D in critically ill patients is multifactorial and may arise from limited sunlight exposure, poor nutritional status, aging, obesity, liver failure, chronic kidney disease, interaction with medications, abnormal gastrointestinal function and effects of fluid resuscitation [21]. Lee et al. [4] speculate that tissues require greater amounts of vitamin D during critical illness, leading to increased conversion of 25(OH)D into the active form 1,25(OH)_2_D, thereby lowering serum levels of 25(OH)D.

We observed a direct association between 25(OH)D deficiency and hospital mortality within 90 days of MICU admission. This association may have multiple causes, given the pleiotropic actions of vitamin D in immunity, endothelial/mucosal function, glucose metabolism and calcium homeostasis. In fact, vitamin D deficiency may help explain many of the varied morbidities frequently observed among critically ill patients, including systemic inflammatory response syndrome, sepsis, organ failure and metabolic dysfunction [4]. In particular, the immunomodulatory actions of vitamin D may explain its observed effects on the prognosis of critically ill patients. In our study, rates of positive blood culture and of MODS were higher among patients with vitamin D deficiency than among those with vitamin D sufficiency; deficient patients also tended to have higher CRP levels. These findings are consistent with recent evidence that vitamin D enhances the innate immune response by inducing production of cathelicidin (LL-37), an endogenous antimicrobial peptide produced by macrophages and neutrophils [22]. This peptide can fight against a broad spectrum of infectious agents, including Gram-negative and -positive bacteria, fungi and mycobacteria [23]. Vitamin D has also been found to down-regulate production of proinflammatory cytokines such as interleukin (IL)-1, IL-2, IL-6, IL-8, IL-12, interferon-γ and tumor necrosis factor-α, as well as T helper 1 cells and B lymphocytes in the adaptive immune system [24], [25]. At the same time, vitamin D up-regulates production of anti-inflammatory cytokines IL-4, IL-5 and IL-10, shifting the overall immune phenotype to a T helper 2 subtype; and it promotes expression of T-regulatory cells, which turn off the adaptive immune response [26]. These cellular and molecular studies suggest that vitamin D deficiency dysregulates the innate immunity system and compromises the ability of critically ill patients to down-regulate the adaptive immune response. These effects may explain the association between vitamin D deficiency and increased mortality observed in our cohort.

Vitamin D is well known as a key participant in the calcium-PTH axis [1], which is responsible for maintaining calcium homeostasis, yet how the axis changes during critical illness is poorly understood. We found hypocalcaemia in 89.4% of our patients, at the upper end of the prevalence range of 15–88% reported for adult patients in the ICU [27], [28]. While hypocalcaemia can be caused by several morbidities frequently found in the ICU, such as sepsis, burns, pancreatitis and rhabdomyolysis, it can also result from vitamin D insufficiency and deficiency. Normally the body avoids hypocalcaemia by boosting secretion of PTH, which increases renal calcium re-absorption and calcium release from the skeleton through bone resorption. The hormone also indirectly increases 1,25(OH)D levels, thereby increasing intestinal calcium absorption. In our cohort, serum 25(OH)D levels did not correlate with levels of ionised or total calcium, suggesting that the hypocalcaemia in our patients had multiple, complex causes. Hypovitaminosis D accounted for only a fraction of hypocalcaemia cases, suggesting that vitamin D supplementation by itself would not correct the problem.

Magnesium is also a major participant in the calcium-PTH axis, and in critical patients, nutritional deficiency and organ dysfunction can lead to magnesium deficiency. This may impair PTH response or result in target organ resistance to PTH, leading in turn to hypocalcaemia. Thus, magnesium deficiency may explain the hypocalcaemia in many of our patients, but we cannot be sure because magnesium levels were not recorded.

Among our patients with secondary hyperparathyroidism, levels of ionised calcium were 25% lower than the normal range observed in patients with sufficient vitamin D. Such reduced levels may reflect severe deficiency of circulating 25(OH)D, age-related impairment of renal 1α- hydroxylation, and compromised calcium absorption.

Approximately 40% of our patients with hypovitaminosis D showed a reduced response to PTH. This proportion is similar to the 60% reported by Nair et al. [29] The potential causes and mechanisms of impaired PTH response to hypovitaminosis D remain unclear. They may include abnormalities in the parathyroid calcium sensing receptor, age-related impairment of renal 1α- hydroxylation, abnormal function of the 1,25(OH)_2_D receptor, abnormalities in the FGF23-Klotho axis and other genetic abnormalities [14]. Magnesium deficiency can also decrease the activity of magnesium-dependent enzymes, inhibiting PTH synthesis and regulation. Whatever the cause, impaired PTH response ironically appears to be associated with better prognosis for critically ill patients. In our study, hypovitaminosis D in PTH-responders was associated with higher APACHE II scores than was the same condition in non-responders. Similarly Nair et al. [29] found PTH-responders to have a higher Simplified Acute Physiology Score (SAPS) II at ICU admission, leading them to speculate that the lack of PTH response may in fact indicate vitamin D sufficiency in tissues. A study of PTH response in elderly with hypovitaminosis D [30] showed that the simultaneous presence of secondary hyperparathyroidism was associated with increased bone turnover and fracture risk, as well as shorter survival. We further found that hypovitaminosis Din PTH-responders was associated with higher 90-day mortality than was the same condition in non-responders.

We did not find a correlation between mortality and PTH in our study, whereas several studies have suggested a direct association between the two variables [31], [32], [33] that was independent of vitamin D status and renal function [31]. Elevated PTH may increase mortality through its effects on cardiac muscle contractility and its ability to promote atherosclerosis and vascular calcification [34]. Elevated PTH may also suppress the immune system, in particular by compromising leukocyte function, which should make patients more susceptible to infection [35]. We speculate that the combined negative effects of hypovitaminosis D and elevated PTH are the most likely cause of increased mortality observed in our PTH-responders with hypovitaminosis D. In fact, the frequent co-occurrence of hypovitaminosis D and impaired PTH response among critically ill patients may reflect the body’s efforts to protect itself from the adverse effects of the altered calcium-PTH-vitamin D axis, and increased expression of calcium sensing receptor in parathyroid glands would be compatible with a more efficient control of PTH synthesis and secretion by low serum ionised calcium.

Our work has several potential limitations. First, the sample size was small, and we did not monitor 25(OH)D, iPTH, and ionised calcium levels over time. Second, our study was conducted in an MICU and cannot be generalised to cardiac, surgical, or other types of ICUs. Third, we did not collect data on levels of 1,25(OH)_2_D, vitamin D binding protein (DBP), or magnesium, so we cannot exclude them as possible confounders. Finally, although our data are consistent with an association between vitamin D deficiency and severity of illness and hospital mortality, we cannot conclude a causative link. Randomised controlled trials to evaluate whether vitamin D supplementation in critically ill patients can improve their clinical outcomes are warranted.

Despite its limitations, our study suggests the need for new lines of research. This study is, to our knowledge, the first to report a correlation between hypovitaminosis D and lower Ig G levels in an ICU population, which should be explored in greater detail in future work. We also found that hypovitaminosis D in PTH-responders is associated with higher APACHE II scores and mortality than was the same condition in non-responders. This raises the intriguing possibility that reduced PTH response in the presence of hypovitaminosis D is a protective mechanism, which should be explored further.

In conclusion, vitamin D deficiency and hypocalcaemia are highly prevalent in the critically ill population, and hypovitaminosis D accounts for only a small number of hypocalcaemia cases. Critically ill patients often show significant dysregulation of the calcium-PTH-vitamin D axis, and vitamin D deficiency is an independent risk factor for prognosis in serious illness. Hypovitaminosis D in PTH-responders is associated with higher APACHE II score and mortality than is the same condition in non-responders. Our findings highlight the complex interactions between PTH and hypovitaminosis D, which merit further study. Our results also suggest that 25(OH)D and iPTH levels should be measured as part of the routine tests performed in the MICU. Future research should examine whether correction of 25(OH)D deficiency improves outcomes for ICU patients.

## Supporting Information

Table S1 **Baseline patient characteristics.**
(DOC)

Table S2 **Characteristics of patients stratified by vitamin D status.**
(DOC)

Table S3 **Characteristics of patients with hypovitaminosis D in the presence or absence of PTH response.**
(DOC)

Table S4 **Logistic regression analysis to analyse factors affecting risk of mortality.**
(DOC)
