# Supplementary material for: Changes in the Calcium-Parathyroid Hormone-Vitamin D Axis and Prognosis for Critically Ill Patients: A Prospective Observational Study
Source: PLoS One. 2013 Sep 20;8(9):e75441. doi: 10.1371/journal.pone.0075441 (PMC3779172; doi:10.1371/journal.pone.0075441)
Supplement: Table S1 — Baseline patient characteristics. (DOC) [file pone.0075441.s001.doc]

Table S1 Baseline patient characteristics

| Variable | Value ^a^ (N=216) |
| --- | --- |
| Male gender, N (%) | 120 (55.6) |
| Age (yr) | 64 (50, 75) |
| APACHE II score | 21 (18, 26) |
| Length of ICU stay (d) | 11.1 (6.8, 23.4) |
| Time on ventilator (d) | 7.15 (3.33, 13.9) |
| iPTH (pg/ml) | 75 (39.4, 128.2) |
| Serum 25(OH)D (ng/ml) | 20.6 (15.1, 33.5) |
| Albumin-adjusted total calcium (mmol/L) | 1.92 (1.79, 2.07) |
| Ionised calcium (mmol/L) | 0.89 (0.75, 0.98) |
| Serum phosphate (mmol/L) | 0.97 (0.61, 1.17) |
| WBC (×10^9^) | 11.3 (8.6, 15.7) |
| Serum albumin (g/L) | 29.8 (27.3, 32.7) |
| Serum creatinine (μmol/L) | 68 (51, 102) |
| Lactate (mmol/L) | 2.2 (1.1, 3.4) |
| 90-day hospital mortality, N (%) | 62 (28.7) |

^a^ Values are reported as median (interquartile range [IQR] 1, IQR3), unless noted otherwise. Abbreviations: APACHE II, Acute Physiology and Chronic Health Evaluation II; ICU, intensive care unit; iPTH, intact parathyroid hormone; 25(OH)D, 25-hydroxyvitamin D; WBC, white blood cell count.
