# Supplementary material for: Changes in the Calcium-Parathyroid Hormone-Vitamin D Axis and Prognosis for Critically Ill Patients: A Prospective Observational Study
Source: PLoS One. 2013 Sep 20;8(9):e75441. doi: 10.1371/journal.pone.0075441 (PMC3779172; doi:10.1371/journal.pone.0075441)
Supplement: Table S2 — Characteristics of patients stratified by vitamin D status. (DOC) [file pone.0075441.s002.doc]

Table S2 Characteristics of patients stratified by vitamin D status

| Variable ^a^ | Sufficient (N=63) | Insufficient (N=58) | Deficient (N=95) | *P* Value |
| --- | --- | --- | --- | --- |
| Age (yr) | 64 (33, 75) | 58 (47, 76) | 67 (54, 74) | 0.297 |
| Male gender, N (%) | 36 (57.1) | 33 (56.9) | 51 (53.7) | 0.886 |
| 25(OH)D levels (ng/ml) | 35.1 (34.1, 40.6) | 22.9 (20.6, 27.2) | 14.3 (13.6, 17.1) | <0.001 |
| APACHE II score | 19 (18, 20) | 22 (17, 26) | 25 (20, 28) | <0.001 |
| Length of ICU stay (d) | 15.2 (10.3, 23.6) | 8.6 (6.9, 20.5) | 10.5 (6.8, 25.3) | 0.113 |
| Time on ventilator (d) | 8 (3.4, 20) | 4.5 (2.8, 11) | 5.3 (3.3, 10.5) | 0.195 |
| iPTH (pg/ml) | 42.1 (27.2, 81.2) | 95.8 (45.9, 121.3) | 95.6 (58.6, 151.2) | <0.001 |
| Albumin-adjusted total calcium (mmol/L) | 1.98 (1.82, 2.11) | 1.99 (1.79, 2.09) | 1.91 (1.78, 2.04) | 0.324 |
| Ionised calcium (mmol/L) | 0.91 (0.84, 0.99) | 0.88 (0.76, 0.96) | 0.82 (0.72, 0.91) | <0.001 |
| Serum phosphate (mmol/L) | 1.01 (0.58, 1.27) | 0.98 (0.51, 1.13) | 0.83 (0.67, 1.07) | 0.75 |
| WBC (×10^9^) | 10.7 (8.6, 14.3) | 11.2 (7, 14.8) | 12.1 (8.0, 21.7) | 0.206 |
| Hb (g/L) | 102 (78, 134) | 97 (91.3, 128.2) | 95 (81.3, 117) | 0.068 |
| Serum albumin (g/L) | 31.2 (27.3, 35) | 29.6 (27.9, 33.4) | 28.3 (25.8, 31.8) | 0.132 |
| Serum creatinine (μmol/L) | 74 (37, 101) | 61 (55, 107) | 68 (47, 101) | 0.829 |
| Ig G (g/L) | 13.43 (9.71, 16.1) | 9.51 (7.32, 12.66) | 9.29 (7.03, 12.21) | <0.001 |
| Lactate (mmol/L) | 2 (1.1, 3.3) | 2 (1.1, 3.2) | 2.3 (1.6, 3.7) | 0.061 |
| CRP (mmol/L) | 69 (37, 124) | 124 (42, 186) | 99 (44, 200) | 0.207 |
| Positive blood culture rate, N (%) | 2 (3.2) | 8 (13.8) | 18 (18.9) | 0.015 |
| MODS, N (%) | 16 (25.4) | 25 (43.1) | 46 (48.4) | 0.013 |
| 90-day hospital mortality, N (%) | 10 (15.9) | 14 (24.1) | 38 (40) | 0.003 |

^a^ Values are reported as median (interquartile range [IQR] 1, IQR3), unless noted otherwise.

Abbreviations: APACHE II, Acute Physiology and Chronic Health Evaluation II; Hb, hemoglobin; CRP, C-reactive protein; ICU,

intensive care unit; Ig G, immunoglobulin G; iPTH, intact parathyroid hormone; MODS, multiple organ dysfunction syndrome;

25(OH)D, 25-hydroxyvitamin D; WBC, white blood cell count.
