# Supplementary material for: Changes in the Calcium-Parathyroid Hormone-Vitamin D Axis and Prognosis for Critically Ill Patients: A Prospective Observational Study
Source: PLoS One. 2013 Sep 20;8(9):e75441. doi: 10.1371/journal.pone.0075441 (PMC3779172; doi:10.1371/journal.pone.0075441)
Supplement: Table S3 — Characteristics of patients with hypovitaminosis D in the presence or absence of PTH response. (DOC) [file pone.0075441.s003.doc]

Table S3 Characteristics of patients with hypovitaminosis D in the presence or absence of PTH response

^a^ Values are reported as median (interquartile range [IQR] 1, IQR3), unless noted otherwise.

| Variable ^a^ | PTH-responders (N=91)  (N=91) | PTH non-responders (N=62)  (N=62) | *P* |
| --- | --- | --- | --- |
| Age (yr) | 65 (51, 76) | 64 (52, 75) | 0.466 |
| Male gender, N (%) | 49 (53.8) | 35 (56.5) | 0.751 |
| 25(OH)D (ng/ml) | 19.2 (13.5, 21.6) | 18.2 (14.2, 21.6) | 0.641 |
| APACHE II score | 26 (21, 28) | 25 (18, 27) | 0.007 |
| iPTH (pg/ml) | 142.3 (105, 166) | 95.8 (56.9, 145.3) | 0.0001 |
| Ionised calcium (mmol/L) | 0.86 (0.74, 0.95) | 0.75 (0.62, 0.82) | 0.0001 |
| Albumin-adjusted total calcium (mmol/L) | 1.92 (1.8, 2.04) | 1.91 (1.79, 2.04) | 0.775 |
| Serum phosphate (mmol/L) | 0.97 (0.52, 1.17) | 0.97 (0.62, 1.13) | 0.842 |
| Hospital mortality, N (%) | 37 (40.7) | 15 (24.2) | 0.033 |

Abbreviations: 25(OH)D, 25-hydroxyvitamin D; APACHE II, Acute Physiology and Chronic Health Evaluation II; iPTH,

intact parathyroid hormone.
