# Supplementary material for: Changes in the Calcium-Parathyroid Hormone-Vitamin D Axis and Prognosis for Critically Ill Patients: A Prospective Observational Study
Source: PLoS One. 2013 Sep 20;8(9):e75441. doi: 10.1371/journal.pone.0075441 (PMC3779172; doi:10.1371/journal.pone.0075441)
Supplement: Table S4 — Logistic regression analysis to analyse factors affecting risk of mortality. (DOC) [file pone.0075441.s004.doc]

Table S4 Logistic regression analysis to analyse factors affecting risk of mortality

| Variable | *P* value | OR | 95% CI |
| --- | --- | --- | --- |
| Vitamin D deficiency | 0.008 | 3.018 | 1.329-6.854 |
| APACHE II score | 0.001 | 1.398 | 1.272-1.537 |
| Age | 0.008 | 1.023 | 1.006-1.041 |
| Lactate | 0.002 | 1.26 | 1.089-1.457 |
| iPTH | 0.959 | 1 | 0.993-1.008 |
| Ionised calcium | 0.971 | 0.953 | 0.073-12.464 |

Abbreviations: OR, odds ratio; CI, confidence interval; APACHE II, Acute

Physiology and Chronic Health Evaluation II; iPTH, intact parathyroid hormone.
